# Clinical Potential of Transcranial Focused Ultrasound for Neurorehabilitation in Pediatric Cancer Survivors

**DOI:** 10.3390/brainsci14030218

**Published:** 2024-02-27

**Authors:** Paul VanGilder, Justin Tanner, Kevin R. Krull, Ranganatha Sitaram

**Affiliations:** 1 Department of Diagnostic Imaging, St. Jude Children’s Research Hospital, 262 Danny Thomas Pl, Memphis, TN 38105, USA; 2 Department of Psychology and Biobehavioral Sciences, St. Jude Children’s Research Hospital, 262 Danny Thomas Pl, Memphis, TN 38105, USA; jtanne98@stjude.org (J.T.); kevin.krull@stjude.org (K.R.K.)

**Keywords:** focused ultrasound, neuromodulation, cognition, neurorehabilitation, pediatric cancer, survivorship

## Abstract

Cancer survivors are at a high risk for treatment-related late effects, particularly neurocognitive impairment in the attention and executive function domains. These can be compounded in pediatric populations still undergoing neural development, which has increased interest in survivorship studies and neurorehabilitation approaches to mitigate these effects. Cognitive training regimens have shown promise as a therapeutic intervention for improving cognitive function. Therapist-guided and computerized training programs with adaptive paradigms have been successfully implemented in pediatric populations, with positive outcomes on attention and working memory. Another interventional approach is neuromodulation to alter plasticity. Transcranial electrical stimulation can modulate cortical surface activity, and cranial nerve stimulation alters autonomic activity in afferent brainstem pathways. However, they are more systemic in nature and have diffuse spatial targeting. Transcranial focused ultrasound (tFUS) modulation overcomes these limitations with high spatial specificity and the ability to target deeper brain regions. In this review, we discuss the efficacy of tFUS for modulating specific brain regions and its potential utility to augment cognitive training programs as a complementary intervention.

## 1. Introduction

Pediatric cancers comprise a varied group of diseases with heterogeneous patterns of occurrence, pathology, treatment, and survival. Advances in diagnostics, pharmacology, treatments, and supportive care have improved overall survivorship rates, but survivors are at high risk for late effects of cancer and treatment-related health burdens that may be chronic and accumulate throughout adulthood. Factors such as age at disease onset, treatment type, and treatment intensity can impact neural development, leading to cognitive impairments that worsen with age and impair quality of life and social, psychological, and economic well-being. This has increased emphasis on survivorship studies to understand mechanisms and improve interventions for these late effects, particularly in regard to cognitive deficits and understanding their neural correlates [1,2,3].

Neurorehabilitation interventions aim to mitigate cognitive deficits that arise due to injury, disease, or neurological disorders. This is especially important in pediatric populations where interventions could serve to normalize brain growth and development over time. Cognitive training regimens and non-invasive neuromodulation techniques demonstrate the ability to treat cognitive symptoms. Pairing neuromodulation with brain training may be a potential avenue for significantly improving cognitive function. Neuromodulation methods have the potential to induce metaplasticity: persistent neuroplasticity that continues after acute interventions [4]. These may augment cognitive therapies by facilitating brain states that are more receptive to rehabilitation by increasing electrical or metabolic brain activity during task performance. However, conventional non-invasive neuromodulation (e.g., transcranial magnetic or direct current stimulation) have drawbacks such as spatially diffuse brain stimulation areas and an inability to penetrate beyond the superficial cortex. Transcranial focused ultrasound (tFUS) has emerged as a promising new neuromodulation approach that may overcome the limitations of existing methods.

Neural structures can be stimulated with tFUS via mechanical energy from ultrasonic waves. It offers a distinct advantage of penetrating bone and soft tissues to reach cortical and subcortical structures with a high degree of spatial specificity (on the mm scale) without the need for invasive surgery. Recent evidence has shown that tFUS is an effective and safe method [5] of non-invasive neuromodulation in animal and human models in cortical sensory, motor, prefrontal, and subcortical areas [6]. The co-registration of tFUS with neuroimaging allows for precise targeting of structures associated with cognitive deficits and may successfully augment cognitive training programs to improve outcomes.

The goal of this manuscript is to illuminate cognitive domains affected in cancer survivors, identify targets that could inform guided interventions, and promote the potential for combined tFUS and cognitive training in these populations. The content will focus on Acute Lymphoblastic Leukemia (ALL), as it is one of the most common pediatric cancers, but cognitive impairment develops across many pediatric and adult cancer diagnoses, e.g., medulloblastoma, extracranial solid tumors, lymphomas, craniopharyngioma, and breast cancer [7,8,9,10]. While ALL, with specific cortical targets and correlated cognitive domains, is a prime candidate to evaluate combined tFUS neuromodulation with established cognitive training programs, translation of care to other diagnoses is promising.

## 2. Cognitive Impairment in Pediatric Cancers

The 5-year survival rates of childhood cancer have significantly improved [11,12,13,14] due to advancements in treatment and supportive care [15] that change the landscape of late effects like cognitive impairment. ALL survivors face long-term morbidity, including chronic health conditions, neurocognitive deficits, and psychosocial impairments [2,3,16,17]. Identification, prevention, and management of treatment-related adverse outcomes are central to follow-up care in the growing population of childhood ALL survivors and improving long-term health-related quality of life. Survivorship in other diagnoses also demonstrates late effects of neurocognitive impairment and concomitant psychosocial disorders [8,18,19,20,21,22].

Long-term ALL survivors demonstrate cognitive impairment in multiple domains linked to treatment modality, intensity, and patient demographics. Domains affected include executive function, processing speed, and attention. In a large cohort study of adult survivors of ALL with established screening methods, survivors exhibit impairment in at least one direct test of executive function, with additional self-reported cognitive dysfunction [23,24]. Historically, childhood ALL was treated with prophylactic cranial radiation, though modern therapies now rely on dexamethasone, intrathecal methotrexate, and high-dose intravenous methotrexate, all of which increase the risk for developing neurocognitive impairments as survivors progress into adulthood [25,26]. Impairments in intelligence, attention, processing speed, executive function, and cognitive ability are consistently observed and exacerbated by radiation dosage, chemotherapy agents, and time since diagnoses [24,27]. Impaired executive functions—such as working memory, fluency, inhibition, and emotional control—are a compounding risk as they are associated with lower educational attainment, unemployment, and emotional distress [28,29]. Impaired cognition can also manifest detrimental bidirectional relationships between sleep, fatigue, and obesity that worsen over time without effective intervention [30,31].

**Figure 1:**
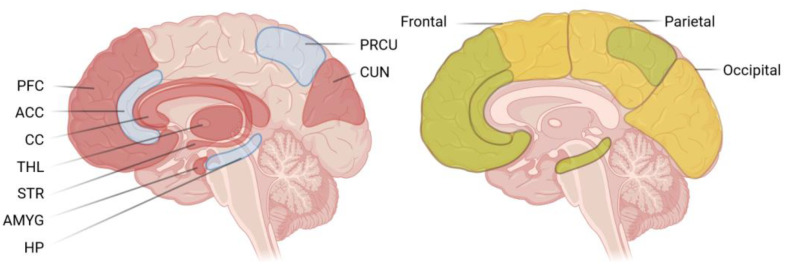
Relevant cortical structures. Left: cortical structures with functional connectivity or volume reduction from ALL survivorship (red) and the structures associated with cognitive impairment in ALL survivors (blue). Right: cortical structures or areas associated with improvements from cognitive training (yellow) and relevant affected structures to target with neuromodulation (green). PFC—prefrontal cortex, ACC—anterior cingulate gyrus, CC—corpus collosum, THL—thalamus, STR—striatum, ANYG—amygdala, HP—hippocampus, PRCU—precuneus, CUN—cuneus. Created with BioRender.com on 3 January 2024.

Cognitive impairments are likely a result of treatments’ effect on brain structure and connectivity. As shown in the left of Figure 1, multiple regions are affected by cancer treatment, and a subset of those regions are associated with cognitive impairment. ALL survivors exhibit reduced volume in brain regions in the frontal, temporal, and parietal lobes as well as the cerebellum, and hippocampus, among other structures. Within these, it is worth noting the reduction in volume of the precuneus, anterior cingulate, frontal subregions, and hippocampus due to their roles in healthy brain network activity [32]. Recent neuroimaging studies suggest that ALL survivors treated with more intensive therapy may have less efficient frontal brain regions. Deviations in connectivity of the right middle frontal gyrus, the left superior occipital gyrus, and the left cuneus are useful predictors of cognitive impairment [33]. Notably, functional magnetic resonance imaging (fMRI) studies have indicated that higher serum concentration of intrathecal methotrexate (MTX) is associated with increased brain activation in dorsolateral prefrontal cortex (DLPFC) during working memory tasks, and diffusion tensor imaging has shown decreased myelin integrity in frontostriatal tracts [26]. Cognitive deficits in ALL survivors have been associated with decreased white matter, and reduced volume in amygdala, thalamus, striatum, and corpus callosum [34,35,36], reduced fractional anisotropy [37], and smaller surface area in prefrontal cortical regions [38]. Some of these differences may be sex-specific, with females exhibiting altered activation patterns in the frontal lobe, precuneus, and cerebellum related to diminished executive function and processing speed [39].

## 3. Existing Therapeutic Interventions for Cognitive Impairment

### 3.1. Cognitive Training Programs

Recent studies suggest that computerized cognitive training offers promising results, especially with early implementation. Standardization and ease of access with computerized care allows for better adherence and comparison between populations. Computerized training strategies with adaptive difficulty promote learning and avoid individualized stagnation.

Investigations into the effect of cognitive remediation programs (CRP) on attention or working memory in pediatric populations have been successful. Researchers modified basic approaches to develop a CRP which specifically addressed attention in survivors of CNS pediatric cancers [40,41,42]. An initial pilot study of this CRP in patients with brain tumor, leukemia, lymphoma, and osteosarcoma found significant improvements in attention and arithmetic compared to controls [41] demonstrating the potential of cognitive training as a successful intervention in pediatric oncology. CRPs vary in treatment status (prophylactic, adjuvant, or remedial), visit counts, course duration, training schedules, training paradigms, outcome measures, and methodological differences. Due to this, direct comparison between different CRP studies is limited, but positive neuropsychological outcomes are typically observed, with evidence that earlier interventions elicit improved outcomes [43,44].

More recent CRPs have continued to produce promising outcomes with in-person training focused on attention and memory skills. Combining a CRP with attention process training produced significant improvements in parent reports of attention and academic achievement, but only 60% of CRP participants completed the regimen [45]. In a similar sample, this approach was applied to address broader psychosocial sequelae by focusing on problem solving skills and compensatory strategies. This CRP’s aim was to generalize attention and memory gains to improve metacognitive skills for everyday life, and saw improvements in social behavior and reading inference [46]. Pediatric cancer survivors undergoing CRPs have shown improved cognitive status, functional independence, and mitigation of fatigue [47] as well as improved mathematics and working memory [48]. These efforts suggest CRPs can mitigate and even prevent neurocognitive late effects but rely on direct and intensive therapist-guided patient interaction—a hurdle to scaling treatment.

Computerized tasks could allow for broader patient access, standardization, and increased adherence. In two pilot studies, at-home computerized cognitive training programs (e.g., Captain’s Log and CogMedRM) demonstrated positive effects on working memory with high participant adherence, and 3-month persistence of effects [49,50]. In a larger study utilizing CogMedRM, pediatric cancer survivors demonstrated lower fMRI activation of frontal lobe regions related to improved working memory, improved processing speed scores, and less executive dysfunction [51]. These neurocognitive benefits persisted at a 6-month follow up assessment [52]. In adult patients, there is evidence that supplementing standard cognitive training with computerized components offers benefits. In post-surgery adult patients with brain tumors, adding computerized interventions to therapist-guided cognitive training improves verbal memory and visual attention [53]. In a similar population, utilizing virtual reality programs in training demonstrated gains in verbal memory and visual coordination [54]. One of the most important aspects that computerized training offers is the ability to include adaptive training strategies, where the task difficulty can be modified based on trial-by-trial performance. ALL survivors who performed an adaptive computerized training intervention showed significant improvements in working memory scores and parent-rated learning problems compared to survivors who performed a non-adaptive version of training [50]. Adaptive training provides enhanced childhood intervention gains for working memory correlated to changes in cortical networks [43] that persist for up to 6 months [55]. Importantly, computerized approaches show a higher degree of patient participation, acceptability, and user satisfaction [50,56].

As training is successful at improving task performance and there is no evidence that cognitive training produces adverse outcomes, a primary concern is in the generalization of any observed improvements to practical benefit [57,58]. Cancer survivors participating in educational and clinical psychology-based cognitive remediation do show improved academic achievement and parent reports of attention [45]. Inpatient cognitive rehabilitation significantly reduced fatigue and improved functional independence in childhood cancer patients undergoing treatment [47]. Educational and attention cognitive therapies produced significant improvements in narrative inference and social cooperation [46]. Reading interventions during radiation treatment of patients with medulloblastoma significantly improved sound awareness scores only. Moreover, fMRI indicated that such interventions have neurophysiological effects: increased activations in ventral occipitotemporal regions and middle/superior temporal gyri [59]. Cognitive training in children from the general population produced improved working memory scores associated with changes in frontoparietal network connectivity to occipital and temporal regions [43]. In older adults and in patients with Alzheimer’s disease with mild cognitive impairment, cognitive training led to increased BOLD activity of the hippocampus associated with improvements in memory [60,61]. Studies in healthy adults also demonstrate that repeated working memory training led to increased BOLD activity in frontal and parietal cortices with improved working memory scores [62]. The generalizability of these outcomes is promising and, as shown in the right of Figure 1, relevant cortical structures overlap with areas affected in ALL survivorship and associated with their cognitive impairment. However, the results from imaging of the neural correlates of cognitive training remain somewhat mixed and dependent on population, disease status, and age. Future efforts should consider the successes listed above to increase the likelihood of gaining generalization: multimodal interventions, such as therapist-guided and computerized training, administration in early treatment status, and adaptive paradigms.

### 3.2. Therapeutic Potential for Non-Invasive Electrical Neuromodulation

#### 3.2.1. Transcranial Electrical Stimulation

Non-invasive Transcranial Electrical Stimulation is a form of neuromodulation that offers accessible approaches to improving cognition or rehabilitating neurological dysfunction. Multiple modalities reliably affect cortical activity, metaplasticity, and behavioral outcomes supporting their status as practical interventions. They are non-pharmacological in nature, safe, generalizable to various populations and pathologies including the developing brain, and produce measurable benefits [63,64]. However, the successes and drawbacks of these approaches must be considered to assess clinical viability for diagnosis- or patient-specific treatment protocols in cancer survivorship-related cognitive impairment.

Transcranial approaches assume the electrical current delivered to the scalp changes the membrane potential in underlying cortical structures, usually targeting the dorsolateral prefrontal cortex (DLPFC). Manipulating electrode polarity, placement, and experimental stimuli modulates neurophysiological and behavioral effects, as outlined in multiple reviews [65,66]. The stimulation modulates neural excitability by polarizing the membrane potential in superficial cortical structures under electrodes. Electrode directionality requires careful consideration, as electrode placement is often bilateral and symmetrical, thereby hyperpolarizing and depolarizing contralateral targets. In symmetrical placements, this produces opposite effects on bilaterally similar structures. In asymmetric placements, the reference electrode is usually placed over the cortex, polarizing unintended and incongruent targets.

Transcutaneous non-invasive cranial nerve stimulation of peripheral trigeminal or vagus cranial nerves modulates the autonomic-related ascending reticular activating system (RAS) in the brainstem [67]. Transcutaneous stimulation can drive the locus coeruleus (LC) and other RAS components in rats and humans [68,69,70]. These superficial cranial nerves connect to the LC and RAS in one or two synapses, offering an access point to modulate activity and create systemic effects relating to plasticity and behavior. Effects on the autonomic nervous system and cognition were studied in invasive cranial nerve stimulation before transcutaneous intervention efficacy was demonstrated without any direct effect on cortex as transcranial stimulation suggests [71].

These neuromodulatory interventions affect neuroplasticity, aiding neurorehabilitation and behavioral outcomes. Transcranial stimulation modulates sodium channels, γ-aminobutyric acid (GABA) levels, glutamate/glutamine concentration (Glx), and brain-derived neurotrophic factor (BDNF) levels [66,72]. GABA, Glx, and BDNF all promote plasticity changes in the cortex that would explain the efficacy of these interventions and how cortical connectivity is altered [73,74]. Similarly, serotonin (5-HT), GABA, and norepinephrine can be modulated by cranial nerve stimulation [69,75], which influence BDNF and cortical plasticity [76,77,78,79,80]. These interventions’ influence on neuroplasticity allows for varied rehabilitative and behavioral benefits.

#### 3.2.2. Behavioral Effects of Electrical Stimulation

Multiple studies have demonstrated that transcranial stimulation may alter network connectivity, producing an assortment of therapeutic benefits in adults and children. Passive transcranial stimulation delivered to the motor cortex often imparts improved motor plasticity [81,82], and cranial nerve stimulation can improve sensory plasticity of tactile rehabilitation and auditory responses [83,84]. Transcranial stimulation of the DLPFC demonstrates benefits in disorders related to executive function or attention impairment as well, such as Autism Spectrum Disorder (ASD) [85], and attention deficit hyperactivity disorder (ADHD) [86]. Cranial nerve stimulation with children with ADHD demonstrates improvements in cognition, mood, and sleep by engaging circuits involved in executive functioning and increasing frontal EEG spectral power [87,88,89]. Exciting results have been observed in adult cancer survivorship as well: better mental health outcomes [90], improved attention [91], and a case study exhibiting improved global cognitive, memory, executive function, and attention scores [92].

### 3.3. Pairing Neuromodulation with Cognitive Training in Cancer Survivorship

Central to our argument is that guided CRPs can be augmented by pairing them with neuromodulation, where learning-induced neural plasticity from repetitive training is enhanced by the plasticity-promoting effects of stimulation. This has been successful in adult and child populations in various populations. Visuospatial cognitive training paired with concomitant transcranial neuromodulation has been shown to enhance gains more than stimulation preceding training, suggesting a window of maximum effect [93]. Parkinson’s patients receiving attention, processing speed, and working memory training with stimulation demonstrated increased motor skill improvements and reduced symptom scores compared to baseline [94]. Children with fetal alcohol syndrome who received DLPFC stimulation alongside cognitive training demonstrated improved attention scores and reported reduced attention deficits compared to sham stimulation [95]. Scores of attention and working memory were improved in unmedicated children with ADHD who received transcranial stimulation [96].

In adult and pediatric cancer populations, transcranial and transcutaneous stimulation studies are limited, but success has been demonstrated. Adult breast cancer survivors who received transcranial stimulation paired with attentional cognitive training demonstrated improved attentional scores and reduced cognitive dysfunction [91,97]. A 2014 case study of a breast cancer survivor with chemo-related cognitive impairment demonstrated the efficacy of DLPFC transcranial stimulation on global cognition, attention, executive function, and memory [92]. The improvement in attention was replicated in a larger study of breast cancer survivors receiving DLPFC stimulation, with improvements in reaction time also observed [98].

Presently, demonstrating the value of cognitive training with the above-presented types of neuromodulations is an active field of research. One study is planned to utilize transcutaneous vagus nerve stimulation to alleviate cancer-related fatigue and depression resulting from treatment of gastrointestinal tumors [99], which could have beneficial effects on general cognitive ability. Also, there are ongoing clinical trials using remote supervised transcranial stimulation in adult survivors of childhood cancer for cognitive impairment amelioration. This study delivers stimulation to the DLPFC in parallel with cognitive training using a battery of executive function assessments to evaluate efficacy [100].

### 3.4. Limitations of Electrical Neuromodulation

As discussed in the previous section, transcranial and transcutaneous methods manifest similar behavioral outcomes, cortical activity changes, affected neurochemical plasticity, and learning improvements. It has been postulated that transcutaneous stimulation may be an alternative explanation for the cognitive, behavioral, and sensory responses observed in transcranial interventions. The latter’s success usually relies on targeting the DLPFC using electrodes at the EEG mapping locations F3 or F4, which lay in the innervated area of the supraorbital trigeminal nerve, and often a reference electrode over the ipsilateral mastoid behind the ear, an area innervated by the vagus nerve [101]. Transcutaneous stimulation patterns are usually biphasic, charge-balanced pulses delivered via electrodes targeting various branches of these nerves and likely drive action potentials in the cranial nerves. Transcranial stimulation may polarize these nerves and increase excitability or also elicit action potentials. The latter is more likely when considering the frequency of stimulation with alternating or random patterns of transcranial stimulation, which often have improved effects. Unintentional stimulation of peripheral trigeminal or vagus cranial nerves with transcranial attempts may indirectly modulate the autonomic reticular activating system in the brainstem. Therefore, many of the transcranial successes could be partially explained by the systemic effects that cranial nerve stimulation has on the autonomic system and downstream neuroplasticity neurochemistry. Potentially, this also explains how transcranial outcomes can be affected by physiological states, wakefulness and mood, i.e., the electrical effect relies on the autonomic state [67,102,103].

While demonstrably effective in neuromodulation of physiology and behavior, the dual mechanistic explanations hinder clinical confidence with specificity or targeting capabilities. Transcranial and transcutaneous neuromodulation methods are useful but bound to their systemic nature and their lack of direct action on specific cortical structures identified in cancer-related cognitive decline. While these methods are productive and practical, transcranial focused ultrasound (tFUS) offers a future direction with precision in mind that could offer exquisite patient-specific care.

## 4. Transcranial Focused Ultrasound

Ultrasound has well-known and wide-ranging clinical imaging applications [104], but has more recently emerged as a promising new tool in translational neuroscience, such as thermal ablation (e.g., of cancerous tissue) [105] or improving permeability of the blood brain barrier for enhanced drug delivery through mechanical interaction of acoustic waves and intravenously injected microbubbles. [106].However, this review focuses on the potential of transcranial focused ultrasound (tFUS) as a method for non-invasively modulating neural activity with better specificity than non-invasive electrical neuromodulation modalities. This novel approach shows great potential for mapping brain–behavior relationships and treating neurological or psychiatric conditions, and this section will discuss the nuances of tFUS as a suitable analogue for neuromodulation, especially when supplementing cognitive training.

The biological effects of ultrasound are highly dependent on sonication parameters, and tFUS can be broadly divided into low-intensity (LIFU) and high-intensity (HIFU) applications based on the delivered acoustic energy. HIFU is typically associated with increased thermal effects and/or tissue ablation, and is generally used in neurosurgery and treating malignant solid tumors [107]. LIFU on the other hand was demonstrated to modulate neural activity over a half century ago [108] but has only recently gained traction as a tool for therapeutic intervention and probing neural circuits and brain organization. Neuromodulation relies on the non-ablative, therapeutic potential of the LIFU subspace.

The guiding principle behind low-intensity tFUS neuromodulation relies on the precise delivery of acoustic energy to a brain region, reversibly altering neural activity without nearing the thermal threshold causing tissue damage. A number of studies have demonstrated that tFUS can be used to enhance or suppress neuronal [109,110,111] and behavioral [112,113,114,115] responses. Although the exact mechanism(s) of action are still an active topic of research, a systematic review of the current literature found it is safe [5].

Using tFUS has distinct advantages over the previously discussed non-invasive approaches, which have drawbacks such as diffuse stimulation area, poor spatial resolution, and limited penetration of the cortical space. Mechanical energy from ultrasonic waves can penetrate bone and soft tissue to target cortical and subcortical structures with millimeter spatial specificity without the need for invasive surgery. Unlike electric and magnetic stimulation approaches, tFUS can be paired with other neuroimaging and recording techniques, such as fMRI, EEG, EMG, and intracortical electrodes (e.g., local field potentials [LFP] and action potentials) to further explore causal brain mapping and offer potential closed-loop feedback. Preliminary studies in animals and humans have demonstrated its efficacy in targeting sensory, motor, and frontal areas of cerebral cortex, as well as deeper structures such as the hippocampus, amygdala, and thalamus.

### 4.1. Results from Animal Studies

#### 4.1.1. Neurophysiological Measures

Early in vitro work in rodent hippocampal circuits demonstrated LIFU could selectively enhance or depress electrical field potential [116,117], affect synaptic transmission [111], and increase the firing rate and induce developing neurons to begin firing [118]. These results were confirmed in vivo, when tFUS stimulation was directed to intact hippocampal CA1 pyramidal neurons and extracellular recordings indicated increased firing rates and evoked oscillatory activity similar to intrinsic hippocampal activity [110]. Stimulation of thalamic regions in rats induced reductions in GABA levels [119] and increases in cortical levels of dopamine and serotonin [120]. These results were important in establishing that tFUS can directly affect localized neuronal activity, even in deeper subcortical regions of the brain. Importantly, indirect effects of stimulation can be observed in downstream cortical areas such as the frontal lobe [120]. Other groups have further demonstrated that tFUS stimulation can selectively modulate cortical neuronal electrical activity, either through suppression [112,121,122] or enhancement [114,123,124] of electric potential, or modulation of oscillatory activity [125,126], depending on FUS intensity [127]. Careful parameter selection can aid in preferentially targeting excitatory or inhibitory spiking responses [128].

Cortical hemodynamics are influenced by FUS stimulation [129], leading to increases in cerebral blood flow [130] or fMRI activations [112,131,132,133]. Stimulation induced whole-brain changes in hemodynamic activity in the mouse cortex, including increased oxygenated hemoglobin (HbO) and decreased deoxygenated hemoglobin (RHb) [129]. The blood oxygen level dependent (BOLD) signal was used to demonstrate excitatory and suppressive effects in rabbit motor and visual cortical activity, respectively [112]. tFUS delivered to somatosensory areas 3a/3b of non-human primates (NHP) elicited similar fMRI activation patterns among regions activated by vibrotactile stimuli and with similar temporal profiles [134].

Further work is needed to understand how stimulation of one region may influence activity within brain networks that subserve behavior. Functional connectivity analyses use correlated BOLD signal changes across regions to estimate interconnected functional networks [135,136,137]. Cortical areas may have their own unique ‘functional fingerprints’ that influence their contribution to cognitive or sensory processing based on connectivity with other regions [138]. It has been suggested that cognitive deficits may be characterized by alterations in network connectivity patterns [139,140,141,142,143], and tFUS may be a useful tool for modulating network activity by precisely targeting important nodes of cognitive networks. Work in non-human primates (NHP) has investigated the effects of sonication to both subcortical and cortical regions within this functional connectivity framework. Sustained, reversible, and dissociable effects of tFUS on functional fingerprints have been demonstrated in NHP amygdala and anterior cingulate cortex (ACC) [131]. Alterations in activity coupling was observed between the amygdala and ACC, and the areas with which each are typically connected, but only when tFUS was applied to the area itself. For instance, tFUS significantly modulated the functional fingerprints of each region in a spatially focalized manner [131]. Disruptions in ACC connectivity patterns may have further implications for cognitive processes [133], as cingulate areas have been implicated in a number of cognitive domains [144,145,146,147,148] and thus may be a potential target for future cognitive neuromodulation studies. Sonication separately targeting the supplementary motor area (SMA) and frontal polar cortex (FPC) led to more uniform activation within the stimulated region, and a gain effect on functionally connected regions, i.e., increased coupling with strongly interconnected areas and reduced coupling with less strongly connected regions [132]. Taken together, these studies reinforce the notion that tFUS stimulation may be a viable tool for focal manipulation of neural networks in the brain. However, it is still unclear if tFUS induces/reduces activity within a region or impacts its responsiveness to inputs from other regions. A notable aspect of these studies [132,133] is a sustained offline effect of tFUS that was detectable up to one hour and 40 min after stimulation, suggesting that the effects may be partly driven by neuroplastic effects of long-term potentiation or depression.

#### 4.1.2. Sensory or Behavioral Responses

Stimulation with tFUS does not just alter neural dynamics but can also have effects on overt motor and sensory behaviors. The most common target for these studies is motor cortex, with movement recorded via EMG or gross observation. One early and compelling study for the efficacy of tFUS for eliciting behavior is from Tufail et. Al., where sonication of the motor cortex in anesthetized rats elicited temporally precise spiking and LFP modulations that were attenuated with tetrodotoxin, implicating that FUS was directly stimulating neuronal output (2010). Stimulation triggered muscle contractions with EMG responses similar to naturalistic muscle twitches. Movement of the transducer over the motor cortex could differentially stimulate isolated muscle groups. Similar results in mouse models were able to reliably and robustly elicit movements of the front legs, hind legs, and tail movements when targeting the motor [115,149,150] and sensorimotor [113] cortices. Interestingly, when tFUS was directed to the visual cortex, neuromodulation (i.e., suppressed BOLD activation and evoked potentials) occurred only when paired with visual stimulation (i.e., sonication alone did not elicit any activity change), indicating a direct effect on sensory processing [112], and that pairing tFUS with behavioral stimuli may be necessary for some tFUS interventions. These contrasting effects on motor and sensory behavior may suggest that tFUS can either elicit action potentials (driving behavior) or suppress excitability in response to sensory inputs.

Involuntary motor movements can be elicited with stimulation of subcortical targets. Eyeball movements and pupillary dilation were observed when superior colliculus, hippocampus, and LC were targets of tFUS in murine brains [113]. Pupillary dilation is a stereotypical indicator of activity in the locus coeruleus, the primary source of norepinephrine in the brain, and with the hippocampus, is a key hub in networks associated with facilitation of sensorimotor behaviors, arousal and attention, and learning and memory (for review [151]), and thus is a prime neuromodulation target for addressing human cognition and driving neural plasticity [76,77,78,79,80].

As the neuromodulation capabilities using tFUS are becoming clearer, it is important to better understand the sustained clinical therapeutic or treatment effects, especially in neurogenesis and neuroplasticity. Promising results on the positive effects of tFUS on working memory and cognitive functioning have been explored in animal models of vascular dementia (VD) and Alzheimer’s disease (AD). Using a Y-maze that tests spatial working memory, VD rats that received tFUS directed at the medial prefrontal cortex (mPFC) exhibited better task performance and improved reward-related working memory impairments compared to sham stimulation [152]. Unfocused, whole-brain ultrasound stimulation of VD and AD mice produced a number of positive effects on cerebrovascular health, neurogenesis, and neuroinflammation, with concomitant improvements in cognitive dysfunction [153]. LIFU stimulation of VD mice upregulated genetic and protein markers related to astrocytes, endothelial cells, and oligodendrocytes. In AD mice, LIFU stimulation suppressed biomarkers of neuroinflammation, inhibiting activated microglia and reducing amyloid-β accumulation. Together, these results provide evidence that tFUS can positively affect the cellular mechanisms in VD and AD pathology. Importantly, behavioral improvements in Y-maze and novel object recognition tests were observed in both models [153]. Earlier work had observed that tFUS directed at mouse hippocampus upregulated BDNF expression [110], and important protein for neuronal health and growth. A PET neuroimaging study provided further evidence of the neuroprotective effects of tFUS related to memory function, detecting increased BDNF levels in the hippocampus and corpus collosum of healthy and VD rats treated with LIFU. Furthermore, VD rats who received LIFU showed remyelination compared to sham. Notably, contextual memory and learning ability of VD rats was improved with tFUS stimulation [154].

Early work has demonstrated the potential for sustained effects of tFUS lasting minutes to hours after stimulation in rat [128,155,156], primate [132], and swine [157] models. FUS-mediated modulation of somatosensory-evoked potentials in anesthetized rats were first demonstrated to last upwards of 35 min [155], suggesting a mechanism for inducing long-term potentiation (LTP) and depression (LTD) of synaptic response. A direct investigation of tFUS-induced plasticity focused on hippocampal networks underlying memory storage and emotional processing. Stimulation of the dentate gyrus induced LTD effects that persisted up to an hour after stimulation [158]. The LTD effects observed were hypothesized to result from modulation of intracellular calcium concentrations [159], a key modulator of hippocampal synaptic plasticity [160,161,162].

### 4.2. Results from Human Studies

#### 4.2.1. Cortical Areas

There have also been investigations into the application of FUS in humans for brain mapping, eliciting behavioral responses, and therapeutic effects. An early modelling study of FUS demonstrated that the acoustic waves targeted at the human somatosensory cortex could effectively penetrate the skull and reach the area with high specificity, altering sensory evoked potentials, spectral content, and phase dynamics [163,164] and eliciting sensory percepts [165,166]. In a pair of early studies, tFUS directed at S1 inhibited neural activity, with reduced amplitude of both short- and long-latency EEG potentials, and modulated sensory-evoked oscillatory dynamics [163,164]. Neural oscillations are thought to underlie long-range communication in the brain [167,168], arising from distributed neural networks that could underlie important aspects of cognitive processes such as perception, memory, and attention [169,170].

Studies have also characterized functional networks during tFUS modulation using fMRI. BOLD responses were assessed concurrently with tFUS stimulation delivered to the primary visual cortex (V1), revealing activations of V1 overlapping with the sonicated region that were not observed during the sham condition [171]. Group-level analysis revealed other regions along the primary visual pathway, including the thalamic nuclei and visual association areas (e.g., precuneus, fusiform, and inferior temporal gyri). These same neural substrates were activated during photic stimulation, suggesting V1 stimulation activated widespread visual circuits. Additionally, fronto-parietal networks, cingulate cortices, and temporal lobe regions, all known to be involved in attentional processing [172,173], were activated. Sonication elicited perceptual phosphenes in subjects, which may have engaged these higher-order cognitive processing networks [174]. Previous studies of BOLD activations in anesthetized animals showed spatially selective activations of the sonicated region; however, these results from awake humans suggest that tFUS stimulation may have broader impacts in the brain, driving concomitant activations in networks involved in perceptual cognitive processing.

Beyond sensory regions, tFUS can also inhibit motor cortical excitability, with observable effects on behavior. LIFU delivered simultaneously with TMS to the human motor cortex inhibited TMS-induced motor evoked potentials (MEPs) and led to reduced reaction times in a stimulus–response task. Importantly, this study also demonstrated that concurrent tFUS and TMS caused no interactions between the ultrasonic and magnetic fields, demonstrating a pathway for more spatially selective exploration of motor cortical excitability [175]. Similarly, Zhang and colleagues demonstrated greater amplitude MEPs during active tFUS stimulation relative to baseline, and improved behavioral inhibition control [176]. Modulating inhibition may be an important therapeutic approach for neurological conditions such as schizophrenia [177], motor dysfunction observed in dystonia [178], or in some aging-related memory impairments [179].

Work in humans has targeted brain regions involved in more complex behavior as well. Areas of the frontal cortex have been stimulated with tFUS with positive effects on emotional states. A pilot study targeted the posterior frontal cortex in patients experiencing chronic pain and observed self-reported increases in mood and decreases in subjective pain [180] that persisted for at least 40 min post stimulation. FUS stimulation of the ventrolateral prefrontal cortex (VLPFC) [181], an area associated with inhibition in cognitive control [182] and emotional processing [183], produced significant increases in global affect relative to placebo controls. Resting state fMRI imaging indicated that functional connectivity was modulated in VLPFC networks and the default mode network 20 min after tFUS stimulation. Decreased connectivity was observed in a network comprising VLPFC, subgenual cortex, orbitofrontal cortex, dorsal anterior cingulate cortex, and entorhinal complex, as well as medial PFC, premotor, and ventral anterior cingulate cortices in the DMN. Increased connectivity was observed between VLPFC and middle frontal gyrus, a region containing the DLPFC, suggesting enhanced inter-region communication in these emotion-regulating regions [184] may underlie the observed increases in mood. Decreases in DMN activity were interpreted as enhancing cognitive control over emotional processing leading to enhanced mood [181]. Importantly, these experiments demonstrate the ability of tFUS to modulate network-level activity that is detectable in fMRI for at least 20 min post-stimulation and may have effects on synaptic plasticity.

#### 4.2.2. Subcortical Areas

In a demonstration of the ability of tFUS to target deep brain regions, stimulation of the human ventro-posterior lateral thalamus inhibited sensory evoked potentials measured by EEG, and attenuated beta and gamma power. Behavioral analysis also indicated that stimulation interfered with discrimination of tactile stimuli to the fingers [185]. These results provide evidence for FUS as means of modulating large-scale networks vis a vis altering cortical oscillatory dynamics by stimulating subcortical structures. Furthermore, previous studies have suggested thalamo-cortical networks may not just represent a passive sensory information pathway, but that thalamus may also play a role as an attentional gating mechanism [186,187,188,189], and lesions to thalamic regions can produce attention and memory deficits (for review see [190]).

The thalamus also serves as a central hub for pain processing, receiving signals from peripheral pain fibers before relaying them to cortical structures that can influence cognitive, attentional, and affective motivational responses [191]. The thalamus has been a therapeutic target for the management of pain, for example, through invasive implantation of deep brain stimulation (DBS) devices [192,193]. However, tFUS may offer a non-invasive therapeutic approach to the treatment of pain, and by extension, possibly pain-related cognitive impairments [194]. To this end, a double-blind controlled study of MR-guided tFUS aimed at the anterior thalamus demonstrated the quantitative antinociceptive effects of stimulation on thermal pain thresholds [195]. Though preliminary, these results suggest tFUS is effective at reducing sensory pain thresholds and may have potential as a noninvasive DBS paradigm.

tFUS was delivered to the globus pallidus (GP) concurrently with fMRI imaging. ROI analysis of bilateral GP, putamen, and thalamus indicated a reduced BOLD signal in the GP and thalamus of the sonicated hemisphere. Offline effects of sonication were assessed using arterial spin labeling imaging, indicating decreased perfusion within the regions of interest. A whole-brain analysis of the BOLD response revealed several foci of reduced BOLD signal in response to GP sonication, including frontoparietal regions, PCC, and Heschl’s gyrus, and a broad decrease in perfusion throughout the cerebrum [196]. These results fit within a framework of pallido–basal–ganglia–thalamo–cortical circuits underlying motor refinement [197] and cognitive functioning [188,198].

Collectively, these studies indicate that tFUS is a promising modality for modulating network-specific and behaviorally relevant cortical activity by targeting subcortical regions that act as relay centers.

### 4.3. Translational and Clinical Approaches

Translational and clinical studies have thus far been limited. In one pilot study, patients with Alzheimer’s disease (AD) received LIFU stimulation to the hippocampal region, as well as pre- and post-stimulation fluorodeoxyglucose-PET neuroimaging. Regional cerebral metabolic rate of glucose increased in frontal, cingulate, and temporal lobes, as well as mild improvements in measures of global cognitive function, possibly through modulation of the hippocampus–prefrontal cortex pathway [199]. In another clinical study of AD patients with depression, LIFU stimulation was delivered to the DLPFC, inferior frontal cortex, bilateral parietal, and precuneus areas in short bursts. A significant reduction in Beck’s depression inventory was observed as well as a normalization of functional connectivity between the insula and the frontal orbital cortex, respectively, relating to the salience and ventromedial networks [200]. In stroke patients with post-stroke cognitive impairment, repeated tFUS was applied to the bilateral frontal lobe and paired with memory and attention training. Patients demonstrated higher BDNF indicating plasticity, augmented EEG response to auditory stimuli, and improved assessments of memory, attention, and general cognitive function [201].

Potential effects of tFUS on neural structures that regulate emotional and sleep qualities have also been investigated. The medial prefrontal cortex (mPFC) is a node within the DMN, and has projections to regions such as the amygdala, hippocampus, and higher-order sensory association areas, making it an important potential target for cognitive amelioration. The mPFC was stimulated using both short-interval (5s) and continuous sonication protocols in a clinical sleep setting [202]. The choice of stimulation type elicited either excitatory or inhibitory responses in EEG spectral power in beta and theta band frequencies, respectively. Since different frequency bands recorded with EEG are thought to coincide with distinct brain functions, particularly during sleep, excitatory or suppressive modulation of specific bands using tFUS may be an approach to targeting brain networks associated with distinct functions in clinically relevant ways.

## 5. On the Promising Potential of Multimodal tFUS

### 5.1. Outlook in Cancer Survivorship

According to neuroimaging evidence from ALL survivors exhibiting cognitive dysfunction, prefrontal or parietal regions may be ideal targets for tFUS. The prefrontal cortex is anatomically and functionally interconnected with a number of cortical and subcortical regions involved in different aspects of executive functioning, such as working memory, behavioral planning and decision making, emotional processing, and top-down control [203,204]. Experimental and clinical studies have identified the DLPFC as a critical region underlying executive function, particularly working memory, using acute non-human primate neurophysiology, fMRI, and lesion studies (for review see [205]), and is highly interconnected with other regions both functionally and anatomically. Frontal–subcortical connections involving the DLPFC, ACC, orbitofrontal cortex, and striatum are involved in the organization and integration of sensory, cognitive, and emotional information guiding cognition and behavior [206]. Furthermore, cognitive training has led to changes in prefrontal activations in these survivors during working memory tasks [51,62], a key cognitive domain impacted by ALL. Future work could pair tFUS targeting prefrontal regions with CRP addressing cognitive deficits observed in ALL, stacking natural learning with induced metaplastic effects.

**Figure 2:**
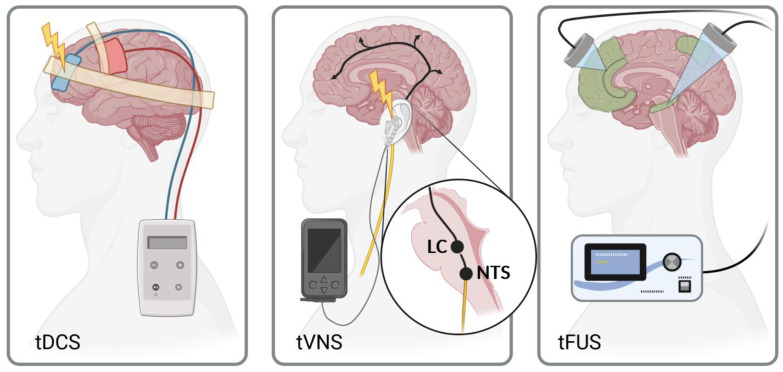
Neuromodulation modalities. (**Left**): transcranial Direct Current Stimulation (tDCS) depolarizes superficial cortical structures under the cathode, increasing potential activity of a large area while the anode may hyperpolarize cortex and suppress activity. (**Middle**): transcutaneous Vagus Nerve Stimulation (tVNS) targets vagus nerve branches (auricular shown) to modulate brainstem activity in the nucleus tractus solitarius (NTS), which affects the locus coeruleus (LC) and indirectly modulates downstream cerebral activity. (**Right**): transcranial Focused Ultrasound (tFUS) can target relevant superficial or deep cortical structures (green, see Figure 1) with high spatial and temporal resolution. Created with BioRender.com on 17 January 2024.

The wealth of relatively recent investigations into neuroanatomical changes in survivorship should be applied to disease- or even individual-specific treatment. Neuromodulation can reliably alter activity in regions where survivors exhibit aberrations such as reduced brain volumes or functional connectivity abnormalities. Similar to electrical neuromodulation, tFUS produces BOLD signal changes when used to stimulate relevant regions of the brain. In addition to the more superficial regions of cortex accessible to transcranial stimulation, tFUS can target deeper cortical structures such as the amygdala, precuneus, or cingulate cortex and elicit effects that extend to their relative downstream connections. (Figure 2) These regions are vital to healthy cognitive processes, and volume reductions observed in survivors are consistently correlated to the impairment of multiple cognitive domains.

### 5.2. Pairing tFUS with Neuroimaging

Co-registering neuroimaging data can leverage the high spatial resolution of tFUS to precisely target cortical or subcortical regions, offering a diagnosis- or patient-specific medicine approach to ameliorating cognitive impairments. Patient-specific neuroimaging data such as structural MRI can be used to precisely position tFUS transducers to accurately, reliably, and repeatably administer stimulation to targeted brain region(s) during cognitive training. Neuroimaging correlates of cognitive dysfunction in different diseases and disorders can be used to identify potential targets for stimulation.

In addition to co-registration for targeting, tFUS can be used in concert with other imaging modalities to further explore the mechanism of action of tFUS and cognition-related activity. High-density EEG offers high temporal resolution of electrical activity in the brain and can identify cortical sources of stimulus-evoked potentials. There is evidence that markers of cognitive deficits in ALL survivors are detectable in dynamics of event-related potentials [207,208,209], and these altered dynamics could be due to changes in network connectivity in cognitive areas of the brain. Utilizing feedback from high-density EEG source localization and biomarker severity can more effectively pair neuromodulation and brain training towards a pronounced focus on patient specificity.

Similarly, tFUS with functional neuroimaging could provide measures of cerebrovascular activity in response to stimulation and training. Pre- and post-stimulation imaging could be used to assess the impacts of paired tFUS and cognitive training on BOLD activity, or functional or effective connectivity. Functional magnetic resonance spectroscopy (fMRS) is an imaging modality that quantifies concentrations of brain metabolites such as neurotransmitters. Neural plasticity during stimulation or cognitive training is believed to rely in part on changes in neurotransmitter and neurotrophic factor regulation. However, it is still unclear how tFUS paired with training would impact these neurotransmitters pathways underlying plasticity. With the right application of compatible hardware, real-time monitoring of fMRI or fMRS activity during tFUS could be used in the development of neurofeedback paradigms of cognitive training to ameliorate deficits.

### 5.3. Safety and Considerations

It is necessary to acknowledge that tFUS is not without limitations. The exact mechanism of action is not thoroughly described, and there is a broad parameter space which must be better characterized to elicit the desired results. For example, pulse repetition frequency, duty cycle, intensity, and carrier frequency have produced divergent results among studies, e.g., excitation or inhibition across different regions, and the reasons have not been fully disentangled. Mechanical pressure on neural tissue altering membrane dynamics (i.e., hyperpolarization) or tFUS preferentially affects certain tissue types (e.g., activating inhibitory GABAergic interneurons [196]. Importantly, the safety of tFUS stimulation has been evaluated in animal and human models and clinical trials in adults, but it has not yet been tested in pediatric populations. Regardless, gross measures of neural activity via EEG, fMRI, or fMRS may help elucidate these mechanisms, differentiate causes for cognitive impairment across diseases or disorders, and provide better framework for further refinement of stimulation protocols for better outcomes.

Safety and efficacy of tFUS in motor, sensory, and emotional domains are well established. Combining the discussed successes of computerized cognitive training, neuromodulation, and their integration, tFUS offers a necessary evolution in capabilities: improving depth abilities and spatial resolution. With recent, albeit sparse, work in adult clinical settings of AD and stroke, there is exciting potential of tFUS incorporation with computerized training in adult cancer survivors as well as pediatric survivors and patients. Targeting DLPFC, precuneus, or the cingulate cortex offers direct action for survivorship support. Consistent direct targeting of regions known to be involved in cognitive domains impaired in cancer survivorship and able to be reliably modulated would accelerate clinical confidence in neuromodulation interventions and offer state-of-the-art patient care in cognitive rehabilitation and related factors.
